# ASSeTS: a systematic review and development of the World Health Organization’s classification system for social isolation and loneliness interventions

**DOI:** 10.1186/s12963-026-00472-7

**Published:** 2026-03-15

**Authors:** D. L. Surkalim, A. Farzana, W. Y. Choo, S. Hussein, P. C. Hébert, V. Welch, E. Tanjong Ghogomu, C. Mikton

**Affiliations:** 1 Department of Social Determinants of Health, World Health Organization, Geneva, Switzerland; 2 Sydney School of Public Health, Faculty of Medicine and Health, The University of Sydney, NSW Sydney, Australia; 3 Department of Social and Preventive Medicine, Universiti Malaya, Kuala Lumpur, Malaysia; 4 Department of Health Services Research and Policy, London School of Hygiene & Tropical Medicine, London, England; 5 University of Ottawa, Bruyere Research Institute, Ottawa, Canada

**Keywords:** Social isolation, Loneliness, Population health, Classification system, Intervention categorization, Policy development

## Abstract

**Background:**

Social isolation and loneliness (SIL) have emerged as critical population health concerns linked to various adverse health outcomes, including cardiovascular disease, stroke, dementia, depression, and premature mortality. However, the absence of a standard categorization for interventions aimed at reducing SIL has impeded consistent comparison, evaluation, and the accumulation of knowledge, affecting evidence-based policy decisions. To address this gap, we developed and empirically evaluated the ASSeTS (Access, Skills, Social engagement, Therapeutic and psychological, Systemic) classification system, a standardized approach for categorizing SIL interventions.

**Methods:**

We conducted a systematic review to identify and evaluate existing classification systems used for SIL interventions. Seventeen databases were searched from inception to September 2023, with no language restrictions. Inclusion criteria encompassed established and widely used reviews with clear intervention categorizations and broad applicability to general population groups. Expert consultations supplemented the systematic review, providing iterative feedback and additional relevant literature missed from the literature search, to inform the development of the ASSeTS classification framework. The developed ASSeTS system was empirically tested by independent experts for clarity, applicability, and reliability, with inter-rater agreement assessed using Fleiss’ kappa.

**Results:**

The review identified 11 studies covering a range of SIL intervention categorization approaches. Based on synthesis and expert feedback, the ASSeTS system was structured into five main categories: Access, Skills, Social engagement, Therapeutic and psychological, and Systemic interventions. Empirical testing yielded moderate inter-rater reliability (κ = 0.419), indicating acceptable usability among expert raters. Higher agreement was found for categories such as therapeutic and psychological interventions, whereas systemic interventions showed lower reliability, suggesting opportunities for future refinement.

**Conclusion:**

The ASSeTS classification system provides a much-needed standardized framework for categorizing SIL interventions, facilitating comparability, rigorous evaluation, cumulative knowledge, and evidence-based policy decisions. Future work should focus on refining less reliable categories, validating ASSeTS across various contexts, and integrating it into global policy frameworks to more effectively address the public health implications of SIL.

**Supplementary Information:**

The online version contains supplementary material available at 10.1186/s12963-026-00472-7.

## Introduction

Social connection–the many ways in which people relate and interact with others [1]–is increasingly recognized as a critical determinant of health across the life course [2]. Extensive research has demonstrated that individuals with stronger social connections experience better physical, mental, and social health outcomes, while those who are socially isolated or lonely face an elevated risk of adverse health conditions [3, 4]. The absence of social connection, manifesting as social isolation and loneliness (SIL), is associated with an increased risk of cardiovascular disease, stroke, dementia, depression, and premature mortality [5, 6]. A recent meta-analysis of 90 studies including adults aged 18 years and older found that social isolation and loneliness can increase the risk of early mortality by up to 32% and 14% respectively [7].

SIL present distinct experiences of deficits in social connection (social disconnection). Social isolation is an objective construct, determined by the number of social relationships and interactions an individual has [8], whereas loneliness is a subjective painful experience, arising from a discrepancy between actual and desired or needed social relationships [9]. There are other forms of social disconnection, including low social capital and support, as well as social negativity; however, this review will focus specifically on SIL.

The pathways through which SIL impacts health can be biological, psychological, and behavioral [1]. Chronic social disconnection has been linked to heightened inflammatory responses, dysregulation of the hypothalamic-pituitary-adrenal (HPA) axis, and impaired immune function, all of which contribute to poor health outcomes and increased mortality risk [10]. An increase in stress may also increase the development and progression of chronic social disconnection through increased rates of depression and anxiety, which can be further exacerbated without strong social connections for support [11]. Additionally, SIL is associated with unhealthy coping behaviors, such as physical inactivity, poor diet, and substance use, further exacerbating health risks [2]. Given these wide-ranging consequences, SIL is increasingly being recognized as a public health priority, with organizations such as the World Health Organization (WHO) and the U.S. Surgeon General’s Office calling for urgent action [12, 13].

Beyond its health impacts, SIL contributes to broader societal inequities, disproportionately affecting marginalized populations, including older adults, individuals with disabilities, low-income groups, and ethnic minorities [1, 14]. These groups often face structural barriers to social participation, such as limited mobility, lack of accessible transportation, and digital exclusion (limited ability or inability to effectively use digital technologies) [15], which compound the risks of social isolation [16]. The effects of SIL are further exacerbated by systemic factors such as urban design, socioeconomic inequality, and discrimination [1, 17]. By framing social connection as a public health priority, we underscore the need for scalable and sustainable solutions that can improve well-being at the population level while reducing the burden of SIL-related health disparities [18].

Despite the growing evidence of the association between social connection and population health there are still prominent gaps within evidence and practice, derived, in part, by non-comparable data and measures [19]. Addressing SIL, therefore, requires a multifaceted approach, incorporating both individual-, relationship-, community-, and societal-level interventions, including policy measures [1, 20]. However, efforts to mitigate SIL have been hampered by limited consensus on how to categorize interventions effectively, owing in part to the lack of methods, descriptors, and theoretical principles for classification, and the wide range of potential intervention types SIL [21, 22]. For example, interventions to target biological or personality factors, behavioral prevention strategies, family-focused or peer and mentorship programs, alterations to physical and social environments, and normative, structural, institutional, and policy system changes [23]. Further, inconsistencies in what can be considered relevant to SIL exist, as there is no universal consensus on what constitutes a SIL intervention. For example, whether changes in SIL must be a primary outcome of an intervention or the proximity of intervention delivery to the individual (e.g., couples counselling may teach awareness and regulation skills that impact factors contributing to SIL in children) [1, 24].

The development of a standardized classification system for SIL interventions, as proposed in this study, is a pre-requisite for successful analysis and comparison of interventions. Greater comparability will ensure that the knowledge generated is cumulative, guiding resource allocation through better gap identification, reduced duplication in evidence generation, promotion of innovation, and informed evidence-based policy and practice for scaling up effective interventions and treatments [2, 25, 26]. This is particularly pertinent for countries and populations with limited resources and financing opportunities, who already experience an inequitable and disproportionate burden from SIL [19, 27].

Current classification systems used in SIL literature are based on different characteristics of the interventions, ranging from mechanism [21, 28, 29], delivery [30, 31], and target population [32]. The lack of rigorous, principle-based guidelines in the field has contributed to the inconclusiveness about the best systems to implement [29]. Examples of existing standardized classification systems include the International Classification of Diseases and Related Health Problems (ICD-Codes) [33] and the International Classification of Health Interventions (ICHI) [34]. ICHI is a reference classification within the WHO’s Family of International Classifications, encompassing all interventions across all sectors of the health system. The classification is built around three areas: (1) target: the entity on which the Action is performed on; (2) action: what is done by an actor to a Target; and (3) means: the processes and methods by which the Action is performed [34]. However, despite ICHI’s broad scope, there are some limitations that restrict its use within the SIL field. For example, ICHI primarily categorizes traditional health interventions and may not adequately capture interventions addressing SIL, which often extend beyond the healthcare sector. Moreover, while ICHI’s general structure provides a comprehensive framework for health interventions, it lacks specificity in guiding the categorization of interventions explicitly designed to address SIL and, importantly, does not easily map to the large body of work already undertaken on SIL interventions, which is less conducive to facilitating future research. To address these issues, this study develops a SIL-specific classification system to be used alongside ICHI – one that draws on the broad scope of ICHI yet reflects and builds on the current SIL literature, incorporating key features of existing frameworks.

To achieve these goals, this study will: (1) identify, review, and evaluate existing classification systems used to categorize interventions that promote social connection and reduce SIL and (2) develop an evidence-based and empirically tested classification system for categorizing SIL interventions that addresses the limitations of existing models, and which can be applied across different settings and populations.

## Methods

This study draws on and adapts methods used in previous efforts [35–37] to develop classification systems and encompasses two major components: (1) a systematic review of existing systems and frameworks, including synthesis of findings through multiple compare and contrast phases, and (2) development and iterative testing of a classification system based on existing literature and expert opinion.

The systematic literature review identified SIL interventions and how different studies categorized or organized interventions, either through explicit organizing principles or presentation of findings. We utilized the WHO Handbook for Guidelines Development [35] and the WHO guidelines to synthesize the results, drawing insights from other papers employing similar methods [36, 37]. We supplemented the systematic review with multistage feedback from a panel of content experts (see Acknowledgements) pre- and post-iterative phases of testing.

This study has been registered on Open Science Framework (10.17605/OSF.IO/KWU63) and conforms to the (United States) National Academy of Medicine’s Psychosocial Interventions for Mental and Substance Use Disorders Framework for Establishing Evidence-based Standards [38] and the Preferred Reporting Items for Systematic Reviews and Meta-Analyses (PRISMA) guidelines (Appendix 1) [39].

### Current intervention classification systems identification

To identify the different methods used to categorize SIL interventions, we sourced existing reviews on SIL interventions from two WHO Evidence and Gap Maps (EGM) [40, 41]. An EGM is a synthesis tool that visually presents the existing evidence and gaps relevant to a specific research topic and the quality of this existing evidence [40]. The two WHO EGMs focus specifically on: (1) in-person interventions for reducing SIL across all age groups; and (2) the EGM on digital interventions for reducing SIL in older adults.

As the EGMs are based on evidence reviewed up to February 2022, an additional updated search using the same search strategy as the original EGMs was carried out to identify more recently published relevant articles for inclusion. We searched for scientific literature published in any language using the following databases: Medline, Embase, Cochrane Central Register of Controlled Trials, PsycInfo, CINAHL, EBSCO, ProQuest, Epistemonikos, Global Index Medicus (LILACS, WPRIM, IMSEAR, IMEMR), Clarivate (Web of Science, KCI-Korean Journal Database, Russian Science Citation Index, SciELO Citation Index), and Scopus. The EGMs’ searches were conducted for literature published from individual database inception until February 2022 [40, 41], while the updated search comprised of literature published from March 2022 until September 2023. Search terms included ‘loneliness’, ‘social isolation’, ‘social support’, ‘improvement’, and types of interventions, combined with other relevant medical subject headings, truncations, and adjacent operators (Table 1).

**Table 1:** Example search strategy (Medline)

	**Search terms**
1	https://protect-ca.mimecast.com/s/7AzeC91Z07iNGZWioN5Pf?domain=loneliness.mp.
2	(loneli* or lonely).tw,kf.
3	*Social isolation/
4	(Social exclus* or social* isolat*).tw,kf.
5	or/1–4
6	*Social support/
7	(Social capital or social cohesion or social contact? or social connect* or social integration or social* interact* or social network? or social participat* or social relation* or social skill*).ti,kf.
8	(Social capital or social cohesion or social contact? or social connect* or social integration or social* interact* or social network? or social participat* or social relation* or social skill*).ab./freq=2
9	((Peer or psychological or psychosocial) adj (counsel* or support*)).tw,kf.
10	Social https://protect-ca.mimecast.com/s/RZo4C0YZGySg1EXIDrnkn?domain=prescribing.tw,kf.
11	*Counseling/
12	Mindfulness/or mindfulness-based interventions/
13	(Meditat* or mindfulness).tw,kf.
14	Psychotherapy/mt, st
15	(Psychoeducation* or psychotherap*).tw,kf.
16	Stress, psychological/th
17	((Improv* or measure*) adj6 (wellbeing or well-being)).tw,kf.
18	((Animal or dog or dogs or pet or pets) adj2 therap*).tw,kf.
19	Or/6–18
20	((Benefit* or change or changes or contribut* or decreas* or develop* or effect or effects or effectiveness or enhance* or evaluat* or experience* or experiment* or impact* or implement* or improv* or increas* or intervention* or method* or outcome* or pilot* or program* or provid* or reduc* or study or support* or system* or target* or technolog* or training or trial or "use of ") adj3 (loneliness or lonely or social isolat*)).ti,kf.
21	((Benefit* or change or changes or contribut* or decreas* or develop* or effect or effects or effectiveness or enhance* or evaluat* or experience* or experiment* or impact* or implement* or improv* or increas* or intervention* or method* or outcome* or pilot* or program* or provid* or reduc* or study or support* or system* or target* or technolog* or training or trial or "use of ") adj3 (loneliness or lonely or social isolat*)).ab.
22	Or/20–21
23	Systematic https://protect-ca.mimecast.com/s/50k_CgZomVUwMg1h2UVBW?domain=review.mp,pt.
24	Meta https://protect-ca.mimecast.com/s/z3ZwCjZrpVUGW64T7DUwx?domain=analysis.mp,pt.
25	(Cochrane or embase or medline or pubmed).ab.
26	Randomized controlled https://protect-ca.mimecast.com/s/dKXdCk8vqViXxLySJJpFA?domain=trial.pt.
27	Controlled clinical https://protect-ca.mimecast.com/s/dKXdCk8vqViXxLySJJpFA?domain=trial.pt.
28	Pragmatic clinical https://protect-ca.mimecast.com/s/dKXdCk8vqViXxLySJJpFA?domain=trial.pt.
29	Randomi*.tw,kf.
30	Placebo.ab.
31	Clinical trials as topic/
32	(Randomly adj2 (allocated or assigned)).ab.
33	Trial.ti.
34	(Group or groups).ab./freq=2
35	((Quasi experiment* or quasiexperiment* or quasi randomi* or quasirandomi*) adj2 (design* or method* or study or trial)).ab,kf.
36	((Before adj5 after) or (controlled adj3 study) or (controlled adj3 trial) or control group* or effect* or evaluat* or (pre adj5 post) or ((pretest or pre-test) and (posttest or post test))).tw.
37	Controlled before-after studies/
38	Interrupted time series analysis/
39	(Time series adj5 (analys* or design* or interrupted or ITS or studies or study or trial)).ab,kf.
40	Or/23–39
41	Animals/not (humans/and animals/)
42	40 not 41
43	5 and (19 or 22) and 42

### Eligibility criteria and study selection

We only included reviews that addressed SIL interventions, excluding primary studies. Studies were included if the review identified more than one type or theme of SIL intervention and contained interventions for a broad general population sample (e.g., community-dwelling older adults but not nursing home residents with dementia) (Table 2). We only included reviews with more than 100 citations at the time of screening as reported by Google Scholar. This threshold was considered a proxy measure for the adoption and relevance of a classification system. The choice of 100 citations being used as the minimum criterion stemmed from a discernible natural cut-off point that was observed between eligible literature (≤ 74 citations vs. ≥ 140 citations). Google Scholar was used to record the number of citations because of its comprehensiveness, allowing us to consistently measure citation numbers from all sources. A final inclusion criterion required reviews to have clear definitions of categories used, if not self-evident (e.g., one-on-one vs. group interventions). This criterion was implemented to address the inconsistencies in how different authors categorized the same interventions, which often led to conflicting data. Greater clarity in categorization definitions was deemed necessary to facilitate data reconciliation. As language and cultural differences would impact the conceptualization and experiences of SIL, no restrictions on language or geography were applied to maximize representation and limit a homogenous account of SIL interventions. At each stage of screening, multiple authors independently screened the articles (DS, CM for the EGM literature searches and DS, AF, WC for the updated search), with disagreements resolved via discussion. Inter-rater agreement was high, with 96% for the EGM-based study selection and 98% for the updated search study selection.

**Table 2:** Examples of excluded studies

Reason for exclusion	Example of excluded study
Non-review (primary) study design	Smith, R., Wuthrich, V., Johnco, C., & Belcher, J. (2021). Effect of group cognitive behavioural therapy on loneliness in a community sample of older adults: a secondary analysis of a randomized controlled trial. *Clin Gerontol*, *44*(4), 439–449.
Focuses on one type/theme of intervention	Vidovic, D., Reinhardt, G. Y., & Hammerton, C. (2021). Can social prescribing foster individual and community well-being? A systematic review of the evidence. *Int J Environ Res and Public Health*, *18*(10), 5276.
Non-general population	McElfresh, J. J., Skiba, M. B., Segrin, C. G., Badger, T. A., Crane, T. E., Crist, J. D., & Thomson, C. A. (2021). Interventions for loneliness among adult cancer survivors: a systematic review and meta-analysis. *J Psychosoc Oncol*, *39*(4), 509–533.
< 100 citations at time of screening	Tong, F., Yu, C., Wang, L., Chi, I., & Fu, F. (2021). Systematic review of efficacy of interventions for social isolation of older adults. *Front Psychol*, *12*, 554,145.
Lack of clearly defined or intuitive categories	Poscia, A., Stojanovic, J., La Milia, D. I., Duplaga, M., Grysztar, M., Moscato, U., Onder, G., Collamati, A., Ricciardi, W., & Magnavita, N. (2018). Interventions targeting loneliness and social isolation among the older people: an update systematic review. *Exp Gerontol*, *102*, 133–144.

### Data extraction and expert panel feedback

Three authors (DS, WC, AF) independently extracted information about SIL interventions from each review, including definitions of social connection and SIL, how the review categorized the interventions (including definitions for categories used and the different (sub)themes used, if not intuitive), a verbatim account of the rationale behind how authors chose to categorize interventions (if included), and whether or not this classification system has been empirically tested in practice. We evaluated the quality of identified classification systems using an adapted version of Ranganathan’s normative principles of classification [42]. Ranganathan’s Prolegomena for classification includes seven canons on which classification systems may be evaluated against: (1) differentiation; (2) concomitance; (3) relevance; (4) ascertainability; (5) permanence; (6) relevant sequence; (7) consistency. Scoring of Ranganathan principles were dichotomous (Y/N). Although comprehensive, we included two other principles deemed to be important and cited in other taxonomy development method literature, but not explicitly included in the list of seven by Ranganathan: conciseness and extensibility [43, 44]. For the purpose of efficiency, we combined conciseness with ascertainability and extensibility with relevance (Table 3). Risk of bias for included studies was evaluated using an adapted version of the Joanna Briggs Institute (JBI) Critical Appraisal Tool for Systematic Reviews and Research Syntheses [45] (Table 4). Disagreements were resolved via discussion. Inter-rater agreement was high, with 90% for the Ranganathan Prolegomena quality assessment and 93% for the risk of bias assessment.

**Table 3:** Ranganathan’s principles of classification canon

Principle	Definition
Differentiation	Categories should differentiate into at least two subcategories.
Concomitance	No two categories should overlap or share the same scope.
Relevance	Categories must be reflective of the context of their content area, with potential for adding further categories in the future.
Ascertainability	Classification system and categories should be easy to use. Categories should be easily distinguishable and understood by name.
Permanence	Identified categories should be comprehensive and exhaustive of their context.
Relevant sequence	Each category should be defined in a relevant hierarchy and intention.
Consistency	The categorisation process should be the same across all contexts, reflected by a Kappa score of ≥ 0.41 as per Landis and Koch.

**Table 4:** Adapted jonna briggs institute systematic review and evidence synthesis risk of bias assessment criteria

	**Criteria**	**Description**
(1)	Review Questions	Is the review question clearly and explicitly stated?
(2)	Inclusion Criteria	Were the inclusion criteria appropriate for the review question?
(3)	Search Strategies	Was the search strategy appropriate?
(4)	Sources of Information	Were the sources and resources used to search for studies adequate?
(5)	Appraisal Criteria	Were the criteria for appraising studies appropriate?
(6)	Critical Appraisal Reviewers	Was critical appraisal conducted by two or more reviewers independently?
(7)	Data Extraction Methods	Were there methods to minimize errors in data extraction?

In addition to the systematic review of literature, a panel of global topic experts were asked for their feedback and recommendations for a SIL classification system (see Acknowledgements). The expert panel was also asked for suggestions for specific classification systems for interventions to address social connection and SIL that they deemed useful or important, which might be missed by the systematic review. Their suggestions and feedback were thematically analyzed and organized by intervention type, and two authors (DS, CM) discussed the panel feedback for relevance and inclusion. Once consensus was reached, edits were incorporated with the results of the systematic review and the resulting framework deemed to be a working model of the proposed classification system.

### Evaluation of proposed classification system

Two authors (WC, AF) evaluated the quality of the proposed classification system using the adapted version of Ranganathan’s normative principles of classification [42–44] (100% inter-rater agreement). To gauge user inter-rater reliability of the classification system, Fleiss’ kappa values were used to determine the inter-rater rater agreement strength between more than two raters [46]. As per standard practice by Landis and Koch [47], a cut-off kappa score of ≥ 0.41 was applied to determine acceptable moderate agreement strength. Using the ‘kappaSize’ function of R (Version 4.3.2) [48] (Appendix 2), the most efficient sample size required for empirical testing of the classification system to achieve a minimum kappa score of ≥ 0.41 is five experts [49]. Initially, eight topic experts from the WHO’s Commission on Social Connection Technical Advisory Group were invited to be part of this testing panel, however four were unable to participate. To complete the five expert panel quota another external expert working with the WHO’s Commission on Social Connection was invited to participate (see Acknowledgements).

The authors identified 11 articles that included specific interventions to address SIL, aiming to include a comprehensive set of interventions that span across the broad spectrum of existing interventions. The expert panelists were provided with the articles and tasked with independently categorizing the interventions in each article using the proposed classification system. To determine inter-rater reliability, panelist responses were uploaded onto SPSS (Version 28.0.0.0) [48] and Fleiss’ kappa values were calculated.

## Results

**Fig. 1:**
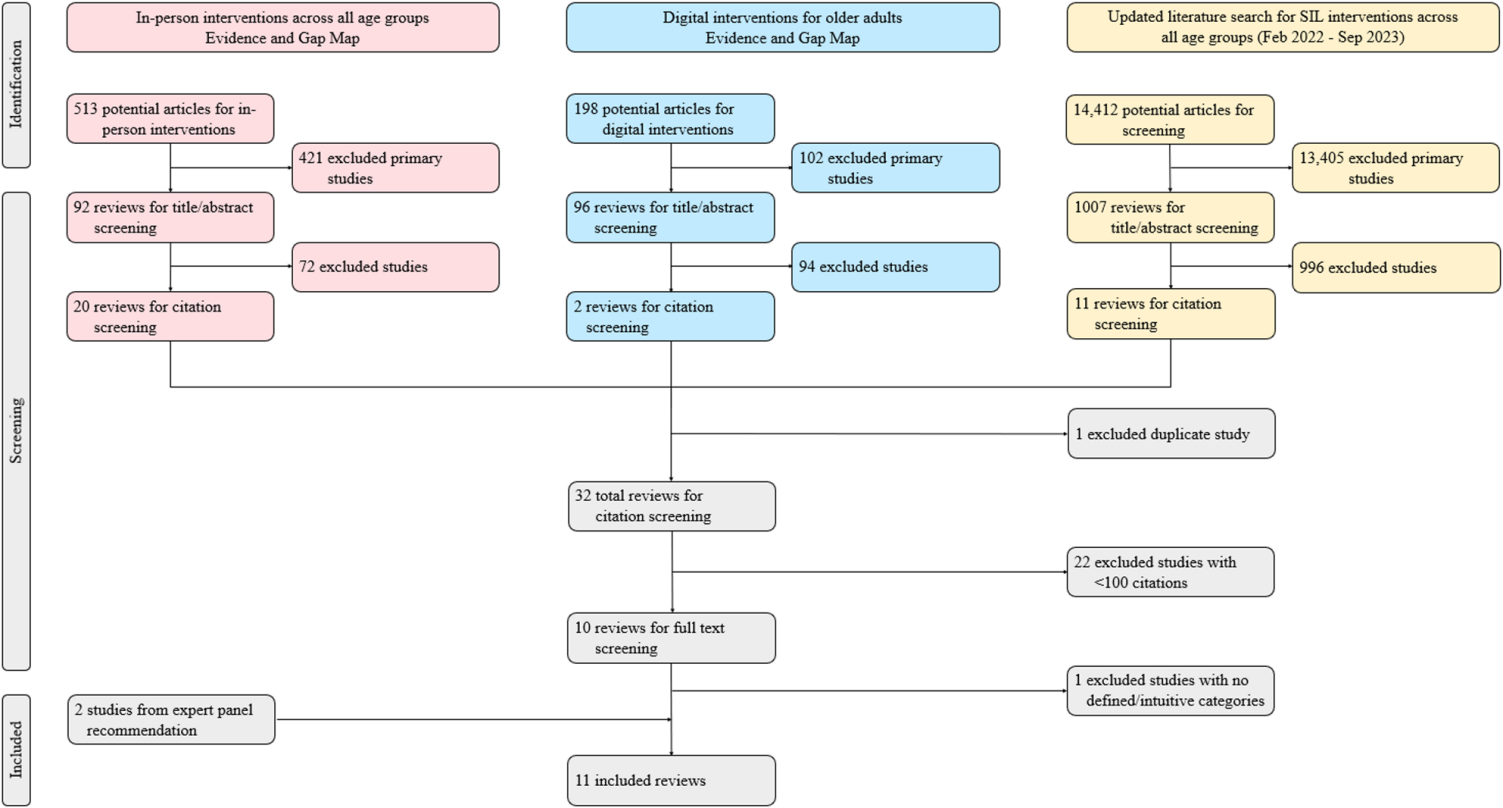
PRISMA study selection flowchart

For the in-person interventions EGM, there were initially 513 articles, out of which 92 were reviews. For the digital interventions EGM, there were initially 198 articles, with 96 being reviews. In the updated literature search, a total of 14,412 articles were identified, including 1007 reviews. Following title and abstract screening and removal of duplicates, 32 reviews qualified for the screening stage requiring at least 100 citations; 10 reviews met the criterion. After screening for the use of clearly defined or intuitive categories, a total of nine reviews were included in the study. An additional two articles were provided by the expert panel for inclusion, resulting in 11 total articles included in the final study (Fig. 1).

The majority of the included studies from the systematic review categorized interventions based on delivery format (how the intervention was administered: e.g., one-to-one vs. group interventions) (*n* = 7) [29, 50–55] and intervention type (the action addressing the causal mechanisms: e.g., psychological therapy, social skills training) (*n* = 5) [28, 29, 52, 53, 56]. Two studies [52, 53] further organized interventions by study design and one study [50] by intervention effectiveness. Nearly half of the identified studies (*n* = 4) [29, 50, 52, 53] used a combination of categorization systems. The two studies provided by the expert panel categorized interventions by either a combination of socio-ecological level (what level of influence the intervention is operating on: e.g., intrapersonal, community, societal) and sector (e.g., transport, housing, education) [2] or solely by intervention type [57], although the latter was limited to psychological interventions (Table 5). Qualitative feedback from the expert panel noted a lack of macro-level interventions, such as vocational training or employment, income, and neighborhood deprivation focused SIL interventions.

**Table 5:** Included studies’ characteristics

Author	Citations^a^	Classification	Rationale for classification	Definition of social isolation	Definition of loneliness	Empirically tested
Cattan (2005)	1403	Delivery format, intervention effectiveness	By effectiveness as per study aim.	Social isolation was considered to be the objective absence or paucity of contacts and interactions between an older person and a social network	Loneliness, or emotional isolation, was defined as the subjective, unwelcome feeling of lack or loss of companionship	No
Cohen-Mansfield (2015)	264	Delivery format	Unspecified	Social isolation indicates having minimal or no contact with other people or with specific types of others	Loneliness is frequently described as a sentiment experienced by a person who defines his/her form or level of relationships as inadequate. Loneliness has both social and emotional dimensions. Social loneliness stems from the absence of a meaningful friendship, whereas emotional loneliness has been described as the absence of an enduring intimate attachment to another	No
Dickens (2011)	903	Intervention type, delivery format, study design	Unspecified	Many authors agree that it is a uni-dimensional concept referring to the lack of social integration… Alternate definitions of social isolation incorporate both ‘structural’ and ‘functional’ social support. Structural social support is an objective assessment of size and frequency, while functional social support is a subjective judgement of the quality or perceived value of emotional, instrumental and informational support provided by others	Loneliness is a subjective concept resulting from a perceived absence or loss of companionship. Social loneliness refers to negative feelings resulting from the absence of meaningful relationships and social integration, whereas emotional loneliness refers to the perceived lack of an attachment figure or confidant	No
Eccles (2021)	140	Intervention type, delivery format, study design	Unspecified	-	Loneliness is experienced when we have fewer social relationships or fewer relationships of sufficient quality than we wish to have	No
Findlay (2003)	682	Delivery format	Based on previous work.	Social isolation, an objective measure of social interaction… people as socially isolated if they had poor or limited contact with others and they perceived this level of contact as inadequate, and/or that the limited contact had adverse personal consequences for them	Social loneliness or emotional isolation, the subjective expression of dissatisfaction with a low number of social contacts	No
Gardiner (2018)	650	Intervention type	Based on thematic analysis.	Social isolation refers to the objective absence or paucity of contacts and interactions between a person and a social network	Loneliness refers to a subjective feeling state of being alone, separated or apart from others, and has been conceptualised as an imbalance between desired social contacts and actual social contacts	No
Hagan (2014)	245	Delivery format	Unspecified	[Social isolation] refers to a lack of engagement with others… ‘knowing relatively few people who are probable sources of rewarding exchanges’	The desolation felt by the loss of someone close is ‘emotional isolation’	No
Hickin (2021)	81^b^	Intervention type (psychological therapies only)	Unspecified	An objective lack of social contact	Loneliness has been defined as a distressing feeling that occurs when there is a discrepancy between desired and achieved social interaction, with the importance of subjective perception in this definition making the concept inherently psychological	No
Holt-Lunstad (2022)	80^b^	Socio-ecological model, sector	Informed by previous use of the socio-ecological model in reviewing other health topics.	An objective indicator of social deficits marked by having few social relationships and roles, and infrequent social contact; a structural indicator of low social connection.	A subjective indicator of social deficits marked by a distressing feeling of aloneness or isolation from others; the discrepancy between one’s actual and desired level of connection.	No
Masi (2011)	1883	Intervention type, delivery format	Not explicit but informed by other systematic reviews.	[Social isolation] reflects an objective measure of social interactions and relationships, whereas loneliness reflects perceived social isolation or out-cast	Loneliness is typically defined as the discrepancy between a person’s desired and actual social relationships	No
O’Rourke (2018)	290	Intervention type	Unspecified	Social connectedness is a positive subjective evaluation of the extent to which one has meaningful, close, and constructive relationships with other individuals, groups, or society indicated by: (1) feelings of caring about others and feeling cared about by others, such as love, companionship or affection and (2) feeling of belonging to a group or community.^c^		No

^a^As of September 2023

^b^Article had less than 100 citations at time of screening, but were added on the basis of expert panellist recommendation

^c^A definition of social connection was provided instead of social isolation or loneliness

### Quality and risk of bias assessment

All included studies scored between 3 and 6 on Ranganathan’s Prolegomena for classification (Table 6). All studies met the criteria for concomitance and relevance, and with the exception of one study [52], all met the criteria for ascertainability. Most included studies also met the criteria for differentiation (82%) and permanence (73%). None of the studies met the criteria for consistency, and only one [2] provided a relevant sequence of organization. The lower compliance with Ranganathan principles may be because the studies included in this review did not specifically aim to produce or use a classification system and thus the authors may not have considered these standards during their study design and execution.

**Table 6:** Included studies’ Ranganathan scores

Author	Differentiation	Concomitance	Relevance	Ascertainability	Permanence	Relevant sequence	Consistency	Total score
Cattan (2005)	No	Yes	Yes	Yes	No	No	No	3
Cohen-Mansfield (2015)	Yes	Yes	Yes	Yes	Yes	No	No	5
Dickens (2011)	Yes	Yes	Yes	No	Yes	No	No	4
Eccles (2021)	Yes	Yes	Yes	Yes	Yes	No	No	5
Findlay (2003)	Yes	Yes	Yes	Yes	Yes	No	No	5
Gardiner (2018)	No	Yes	Yes	Yes	Yes	No	No	4
Hagan (2014)	Yes	Yes	Yes	Yes	No	No	No	4
Hickin (2021)	Yes	Yes	Yes	Yes	No	No	No	4
Holt-Lunstad (2022)	Yes	Yes	Yes	Yes	Yes	Yes	No	6
Masi (2011)	Yes	Yes	Yes	Yes	Yes	No	No	5
O’Rourke (2018)	Yes	Yes	Yes	Yes	Yes	No	No	5

All but one included studies [28, 29, 50–57] scored 3–7 on the adapted JBI tool for systematic reviews and evidence synthesis [45] (Table 7). The final study [2] scored 0 as it was a narrative review. However, as this study was provided by the expert panel, the authors chose to include this study in this systematic review after further examination and screening for relevance, noting the low JBI tool score was predominantly due to the format of the study. Excluding this one study, all other studies met criteria 1 (review questions), 3 (search strategies), and 4 (sources of information). Nine studies [28, 29, 50–54, 56, 57] met criterion 2 (inclusion criteria), with the remaining study [55] not meeting this due to the partial narrative review format of the study. Half of the studies [28, 51–53, 57] reported clear appraisal criteria (criterion 5). For both criteria 6 (critical appraisal reviewers) and 7 (data extraction methods), only three studies [52, 53, 57] reported sufficiently clear methods to meet these criteria, with nearly half of the studies [28, 29, 50, 55, 56] providing inadequate methodological details.

**Table 7:** Included studies’ adapted Jonna Briggs Institute systematic review and evidence synthesis risk of bias assessment

Author	Review questions	Inclusion criteria	Search strategies	Sources of information	Appraisal criteria	Critical appraisal reviewers	Data extraction methods	Total score
Cattan (2005)	Yes	Yes	Yes	Yes	No	Unclear	Unclear	4
Cohen-Mansfield (2015)	Yes	Yes	Yes	Yes	Yes	No	No	5
Dickens (2011)	Yes	Yes	Yes	Yes	Yes	Yes	Yes	7
Eccles (2021)	Yes	Yes	Yes	Yes	Yes	Yes	Yes	7
Findlay (2003)	Yes	Yes	Yes	Yes	No	No	No	4
Gardiner (2018)	Yes	Yes	Yes	Yes	Yes	Unclear	No	5
Hagan (2014)	Yes	No	Yes	Yes	Unclear	Unclear	Unclear	3
Hickin (2021)	Yes	Yes	Yes	Yes	Yes	Yes	Yes	7
Holt-Lunstad (2022)	No	No	No	No	No	No	No	0
Masi (2011)	Yes	Yes	Yes	Yes	Unclear	Unclear	Unclear	4
O’Rourke (2018)	Yes	Yes	Yes	Yes	No	No	Unclear	4

### Proposed classification system

**Fig. 2:**
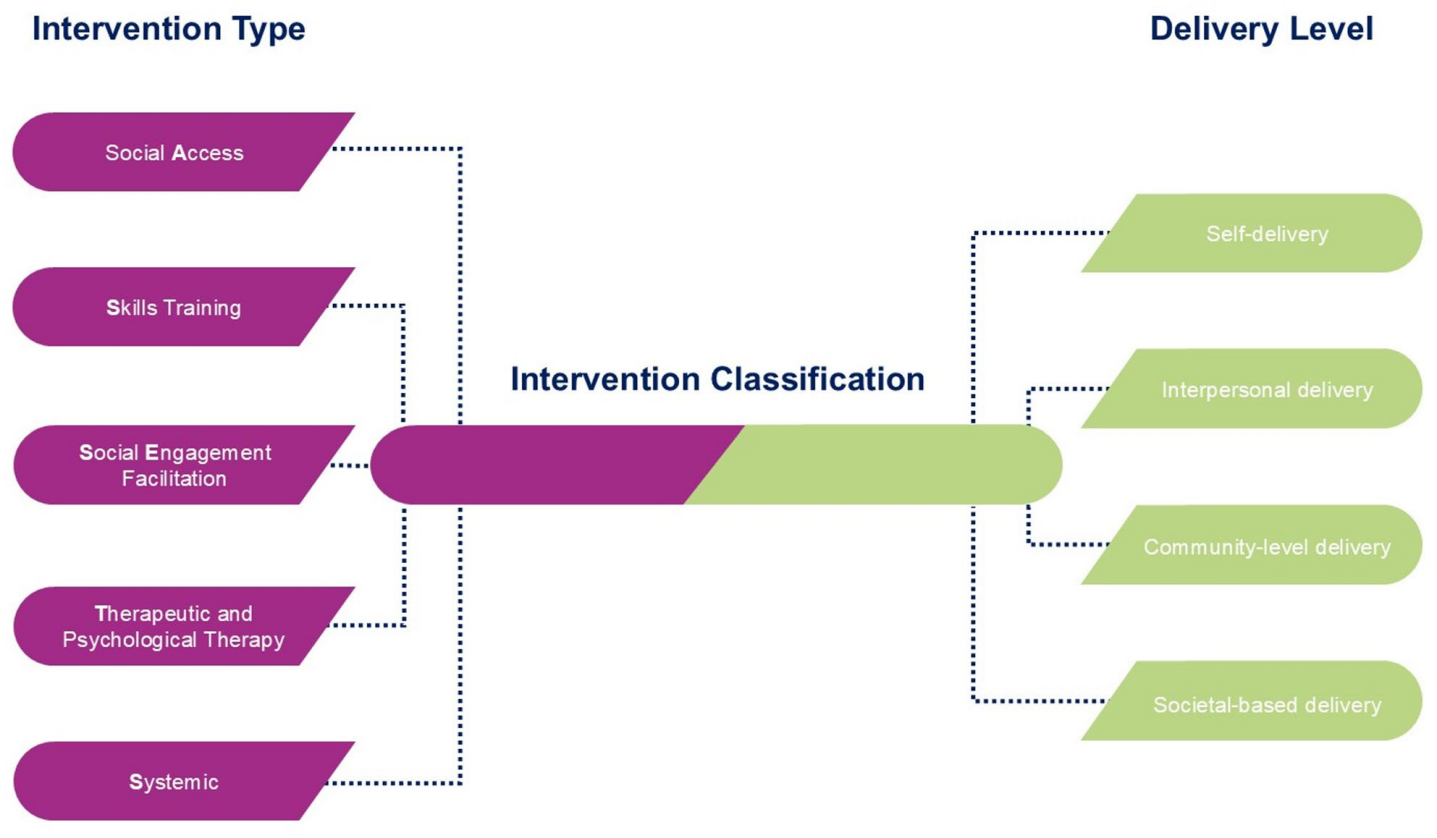
ASSeTS classification system visualization

The proposed classification system combines the most common organizing principles identified by the systematic review (delivery format and intervention type), as this exercise aimed to be informed by the most widely used methods available. Based on the feedback from the expert panel, we initially aimed to incorporate the socio-ecological model [58] into our proposed classification system. However, we found that the multiple levels of the socio-ecological model (such as individual, interpersonal, community, and societal levels) overlapped significantly with intervention delivery format. This overlap risked creating confusion and redundancies during categorization, so we decided to merge the two levels of classification into one. The proposed classification system will therefore be comprised of two major components: intervention type, supplemented by the socio-ecological level of delivery (Table 8; Fig. 2).

**Table 8:** ASSeTS classification system

Intervention Type	Presumed primary mechanism/s of action	Definition	Example(s)
Social Access	Provision of opportunities	Interventions that provide or increase structural opportunities for participants to engage in social interactions	Public transport Social media platforms Community gardens
Skills Training	Ability to manage quality relationships and interactions	Interventions that aim to teach or improve skills related to relationship building or maintenance	Social skills/resilience training Leisure skills development Internet/technological skills upskilling
Social Engagement Facilitation	Fostering social interaction	Interventions that actively fosters consistent or regular interactions (human or otherwise)	Support groups Animal therapy
Therapeutic and Psychological Therapy	Ability to manage quality relationships and interactions	Interventions that aim to change maladaptive thinking patterns and/or improve self-efficacy skills	Cognitive behavioural therapy Mindfulness therapy
Systemic	Complex, e.g., knowledge and awareness of social connection	Systemic changes to operational or knowledge management of SIL	Public awareness campaigns Grants for social connection research
Multicomponent	-	Interventions with any combination of the five other mechanisms	
Other	-	Other intervention mechanisms not otherwise specified	
**Socio-ecological level of delivery**			
Self-delivery		Self-administered interventions	Self-guided therapy Self-taught learning
Interpersonal delivery		Interventions administered by professionals, volunteers, or others in an individual’s social network	Support groups Leisure/hobby groups Family therapy
Community based delivery		Interventions administered by organizations, services, or facilities, within a community or organizational setting (e.g., schools, hospitals, workplaces etc.)	Accessible/affordable transport Neighbourhood/housing design Walkable infrastructure Workplace policies
Societal level delivery		Interventions administered that target the macro-level factors affecting broader society	Policy Research Public Education Campaigns

The intervention type is defined as the primary process or method used by a specific SIL intervention to reduce levels of social isolation and/or loneliness or improve SIL outcomes. For the purposes of this classification system, the intervention type refers to the process or method used by the intervention to affect one of two causal mechanisms of SIL: increasing protective factor(s) or decreasing risk factor(s). This is similar to the Means component of ICHI [34]. There are five specified major intervention types, with additional options for more complex interventions. These five types are: 1) social access; 2) skills training; 3) social engagement facilitation; 4) therapeutic and psychological; and 5) systemic. Additionally, there are ‘multicomponent’ and ‘other’ options available for interventions that involve multiple components or do not fit into the specified categories. Thus, we propose the new ASSeTS (Access, Skills, Social engagement, Therapeutic and psychological, Systemic) classification system of SIL interventions.

Social access interventions aim to increase opportunities for participants to engage in social interactions, with the primary mechanism of action being the provision of opportunities. Social access interventions will focus more on external infrastructure, such as interventions that target the built environment [59], social prescribing [60], and online forums facilitating interaction with others [61].

Skills training interventions aim to teach or improve skills related to relationship building or maintenance, outside the realm of intrapersonal cognitive psychological therapies. The primary mechanism of action is the promotion of individuals’ abilities to manage quality relationships and interactions. Skills training interventions will focus more on interpersonal skills development, such as social skills training [29], internet training [62], and leisure skills development [63] (e.g., sports training).

Social engagement facilitation interventions aim to directly provide participants with consistent or regular interactions. Unlike social access interventions, the primary mechanism of action involves fostering social interactions through direct provision of interactions, in comparison to provision of opportunities for interactions to occur. Social engagement facilitation interventions will focus more on external intra-personal engagement, such as peer-to-peer support groups [64], home visiting programs [50], and animal companionship [65].

Therapeutic and psychological interventions aim to alter maladaptive thinking patterns, increase self-efficacy, and better regulate emotions. Similar to skills training interventions, the primary mechanism of action involves promoting individuals’ ability to manage quality relationships and interactions. However, therapeutic and psychological interventions will focus more on intrapersonal processes with the help, for instance, of behavioral or psychodynamic interventions: e.g., cognitive behavioral therapy and reminiscence therapy [57].

Systemic interventions aim to influence upstream, societal-level determinants of SIL, such as lack of or inadequate laws, norms, policies related to SIL, inadequate public understanding and awareness, inadequate funding for addressing SIL, and poor social infrastructure. Examples include the development of national policies surrounding the design of neighborhoods [66], education campaigns for raising awareness and destigmatization of SIL [67], and greater provision of resources surrounding best SIL practices [67].

In addition to intervention type, we propose the use of socio-ecological level of delivery as a supplementary categorization feature. Although the EGMs [40, 41] primarily categorized SIL interventions by socio-ecological level, this method does not account for the potential of interventions of the same type to be delivered on multiple socio-ecological levels, or the ability of different interventions to target different and multiple determinants of SIL and health. Thus, we propose the more clearly defined and easily differentiated intervention type categories to be the primary classification system and delivery level to be an additional identifier for ease of use and management. Self-delivery interventions are those that are self-guided. Interpersonal delivery interventions are those that are administered by professionals, volunteers, or others in an individual’s social network [40]. Community based delivery interventions are those administered through organizations, services, or facilities within a specific community of organizational setting. Societal level delivery interventions are those that are administered on a wider society level to target macro-level factors of SIL. For example, a social access intervention (self-delivery) (e.g., video calling platforms) [68] vs. a social access intervention (community based delivery) (e.g., communal spaces in a local neighborhood) [69].

### Empirical testing of ASSeTS

Using the adapted version of Ranganathan’s normative principles of classification to evaluate quality [42–44], two authors (WC, AF) involved with the literature review and data extraction, but not directly involved in the development of the classification system, rated the ASSeTS system. They were selected for their familiarity with the existing literature on classification systems, enabling informed comparisons, while maintaining adequate distance from the development of ASSeTS to ensure unbiased ratings. The quality reviewers agreed on assessments for all Ranganathan principles. Both reviewers deemed ASSeTS met the criteria for six Ranganathan principles (differentiation, concomitance, relevance, ascertainability, permanence, consistency) and did not meet criteria for one (relevant sequence).

Overall, the expert testing panel achieved a kappa score of 0.419 (95% CI = 0.333–0.505), achieving a moderate level of agreement using the ASSeTS classification system (Table 9) [9]. For individual categories, the expert panel achieved moderate agreement for skills training (κ = 0.713; 95% CI = 0.526–0.899), social engagement facilitation (κ = 0.473; 95% CI = 0.286–0.660), and therapeutic and psychological interventions (κ = 0.730; *p* < 0.001; 95% CI = 0.544–0.917), indicating greater likelihood that these types of interventions would be more easily agreed upon when using the ASSeTS system. The expert panel achieved fair agreement for systemic interventions (κ = 0.326; 95% CI = 0.139–0.513), and only slight agreement for social access (κ = 0.120; 95% CI=−0.067−0.307), multicomponent (κ = 0.206; 95% CI = 0.019–0.392), and ‘other’ interventions (κ = 0.056; 95% CI=−0.131−0.243).

**Table 9:** ASSeTS expert testing panel inter-rater reliability outcomes

Study	Rater 1	Rater 2	Rater 3	Rater 4	Rater 5
Bartlett (2019)	Therapeutic and Psychological Therapy	Therapeutic and Psychological Therapy	Therapeutic and Psychological Therapy	Other	Therapeutic and Psychological Therapy
Chan (2017)	Multicomponent	Social Engagement Facilitation	Social Engagement Facilitation	Skills Training	Social Engagement Facilitation
Czaja (2017)	Multicomponent	Social Access	Social Access	Social Engagement Facilitation	Multicomponent
Dingle (2022)	Social Engagement Facilitation	Social Engagement Facilitation	Social Engagement Facilitation	Social Engagement Facilitation	Social Engagement Facilitation
Nazari (2021)	Multicomponent	Skills Training	Skills Training	Skills Training	Skills Training
Ose (2023)	Skills Training	Skills Training	Skills Training	Skills Training	Multicomponent
Quinn (2021)	Skills Training	Skills Training	Skills Training	Skills Training	Skills Training
Razani (2018)	Multicomponent	Social Engagement Facilitation	Social Access	Social Engagement Facilitation	Social Access
Robinson (2013)	Social Engagement Facilitation	Social Access	Social Engagement Facilitation	Other	Social Engagement Facilitation
Rodríguez Romero (2020)	Systemic	Multicomponent	Multicomponent	Multicomponent	Multicomponent
Timmermans (2020)	Systemic	Systemic	Other	Other	Systemic

Category	κ	Standard Error	z	Sig.	95% Confidence Interval	
					Lower Bound	Upper Bound
Overall	0.419	0.044	9.549	< 0.001	0.333	0.505
Social Access	0.120	0.095	1.259	0.208	−0.067	0.307
Skills Training	0.713	0.095	7.473	< 0.001	0.526	0.899
Social Engagement Facilitation	0.473	0.095	4.961	< 0.001	0.286	0.660
Therapeutic and Psychological Therapy	0.730	0.095	7.660	< 0.001	0.544	0.917
Systemic	0.326	0.095	3.419	< 0.001	0.139	0.513
Multicomponent	0.206	0.095	2.156	0.031	0.019	0.392
Other	0.056	0.095	0.591	0.554	−0.131	0.243

## Discussion

Given the established impact of SIL on individual and population health, including its association with increased risks of cardiovascular disease, stroke, dementia, depression, and premature mortality [5, 6], there has been a growing emphasis on developing and implementing interventions to address SIL. However, existing efforts to categorize and evaluate these interventions have remained fragmented and inconsistent, leading to challenges in comparing effectiveness, ensuring cumulative knowledge, guiding policy decisions, and scaling evidence-based approaches [21, 23]. Previous classification systems have categorized SIL interventions using disparate frameworks, focusing on mechanism of action, delivery format, or socio-ecological level [22, 28]. While these approaches have provided valuable insights, they lack a unified structure that can effectively map and evaluate the diverse range of SIL interventions across disciplines. To address these limitations, this study developed ASSeTS (Access, Skills, Social engagement, Therapeutic and psychological, Systemic) – a comprehensive, structured, and empirically tested classification system that integrates key features of existing frameworks while addressing their limitations. By providing a standardized approach for organizing SIL interventions, ASSeTS enhances the comparability of interventions, supports evidence-based policymaking, and facilitates more effective resource allocation, particularly in low-resource settings where the burden of SIL is disproportionately high [18, 20].

Nearly half of the studies [29, 50, 52, 53] included in this systematic review used a combination of categorization systems, with the most common combination being intervention type and delivery format. The lack of consistency between different classification systems may be a result of the lack of standardized methods or standards in the field for the development of a classification system of interventions. Inconsistencies within the studies in this systematic review may also result from a lack of intentional efforts by the respective authors to create or use a classification system of interventions, as the ad hoc organization of interventions in the included studies were not the primary aim of each study. General observations from the systematic review also highlight a dearth of upstream SIL interventions. Expert panel feedback confirmed this observation, noting a lack of research and evaluation efforts for macro level interventions, such as income-based interventions (for example vocational training and employment) or interventions addressing neighborhood deprivation.

### Framework

When initially analyzing the literature from the systematic review the authors aimed to further categorize classification systems by guiding principles. The TIDieR framework [70] was initially identified to determine specific classification system components, however the framework was found to be less applicable as it primarily focuses on identifying components of individual interventions, rather than entire systems or categories of interventions. Therefore, the framework’s checklist items were found to be less relevant: items regarding specific rationale and goals of the intervention, required intervention materials, procedures, providers, infrastructure, dosage, duration, intensity, tailoring, modifications, and fidelity [70]. As an alternative approach, we developed the ASSeTS classification system using an adapted version of the Psychosocial Interventions for Mental and Substance Use Disorders Framework for Establishing Evidence-based Standards [38]. Although the National Academy of Medicine’s framework is also based on individual interventions, specifically psychosocial interventions, the framework specified only three main concepts to outline: (1) intervention; (2) how the intervention might affect change; and (3) outcomes. The ASSeTS classification system (Table 8) outlines the different intervention types (intervention), presumed primary mechanism of action and definition (how the intervention might affect change), with all categories having mutual outcomes of either increasing SIL protective factor(s) or decreasing SIL risk factor(s) (outcomes).

It is possible that certain intervention types may be more likely to target specific determinants of social isolation, loneliness, or both. For example, the presumed primary mechanism of action for therapeutic and psychological interventions is the ability to manage quality relationships and interactions. Therefore, interventions to address this specific mechanism of action are likely to focus on determinants of loneliness, which are largely subjective. Although it may be possible to map intervention types and respective mechanism(s) of action to social isolation, loneliness, or both, due to the wide range of possible, and developing, interventions, it is likely that there exist interventions to address both SIL across all intervention types. Thus, it is recommended when using the ASSeTS classification system that users determine what the primary mechanism of action that an intervention is attempting to address when considering which intervention type would be best applicable.

### Strengths and limitations of this study

To ensure that the classification system was reflective of both current literature and stakeholder needs, we undertook a systematic review and expert panel consultations to inform and consolidate the ASSeTS classification system. This approach maximized the scope of our information search by incorporating both published literature and end-user opinions. Additionally, the members of the expert panel consultations comprised leaders in the field of social connection practice and research, drawn from various divisions of the WHO’s Commission on Social Connection. We empirically tested the ASSeTS classification system to ensure the accuracy, repeatability, and useability of the classification system. Empirical testing of the ASSeTS classification system yielded a moderate kappa score (κ = 0.419). Despite meeting the threshold for acceptable agreement (κ ≥ 0.41), this is considered relatively low. This could potentially be attributed to language or cultural barriers. Despite the efforts to ensure regional diversity with the testing panel, the articles provided for testing were only in English. Language or cultural nuances in the interpretation of interventions or social (dis)connection-related terms may have led to some confusion in the categorization process [27, 71]. Additionally, the accurate application of ASSeTS may also be reliant on having some clinical experience or knowledge, particularly for identifying therapeutic and psychological interventions. Non-clinical users may not recognize these interventions accurately, resulting in misclassification. Further, the inclusion of a multicomponent or ‘other’ intervention type option may prompt users to select these options out of uncertainty and choice overload, rather than committing to a specific intervention type [72].

The ASSeTS classification system was assessed to have met all Ranganathan principles except for ‘relevant sequence’. This was an intentional decision as we opted to prioritize the use of a mnemonic acronym to name and organize the classification system, for easier retention and adoption [73]. Ideally, the authors would have organized the different categories in an order similar to the socio-ecological model, with an intrapersonal-interpersonal-group-community-society adjacent order [58], however usability considerations took precedence in this decision-making process.

As there is a lack of standardized methods or best practice guides for the development of classification systems for interventions [37], we used adapted versions of existing methodologies from a range of sources. Further, we used evidence-based decision-making, using established processes from other similar fields to best inform our procedures, ensuring robustness in our methodology. We recognize that this classification system is still at an early stage and requires further testing and refinement. Because the period for the literature search ends in September 2023, updated searches and reassessment of citation metrics would also be required for future iterations or refinements of the classification system.

### Future implications

As the ASSeTS classification system is the first classification system for SIL interventions which has been developed using rigorous empirical testing, we encourage others to use it both for research and in clinical practice with a view to further refining it. Use of the ASSeTS classification system may also highlight gaps in literature and practice regarding the lack of specific intervention types in addressing SIL, which may further inform future investments in research going forward. The ASSeTS classification system may also be used to identify non-traditional, or non-traditionally health, interventions that may impact SIL, by considering the intended outcomes and mapping to the primary SIL mechanism(s) of action that could be impacted. This may contribute to greater innovations and considerations for co-benefits. We acknowledge that the ASSeTS classification system produced by this study is an initial version of the classification system. Future revisions to the ASSeTS system should include an updated literature search and assessment of classification system adoption and influence. The addition of new and emerging intervention types should be considered as the field of SIL research develops.

## Conclusion

Social isolation and loneliness, as two important forms of social disconnection, are vital concerns for population health, with well-documented links to increased risks of cardiovascular disease, dementia, depression, and premature mortality [5, 6]. Although there is growing recognition of SIL as a determinant of health, attempts to mitigate its effects have been hampered by the absence of a standardized approach to categorizing interventions. Current classification frameworks have been fragmented and inconsistent, complicating efforts to compare interventions, assess their effectiveness, and guide policy and funding decisions [22, 28].

To address this gap, this study developed ASSeTS (Access, Skills, Social engagement, Therapeutic and psychological, Systemic) – the first formalized and empirically tested classification system for SIL interventions. By integrating multiple dimensions of intervention categorization, ASSeTS provides a structured and comprehensive framework that enhances the comparability, evaluation, and scalability of SIL interventions across research and practice and will promote cumulative knowledge in the field. The development and empirical validation of ASSeTS mark a significant advancement in the field, offering a tool that can be used by researchers, policymakers, and practitioners to better design, implement, and assess interventions.

Future research should focus on further refining the ASSeTS classification system through broader validation efforts, cross-cultural testing, and real-world application in different policy and healthcare settings. Additionally, integrating ASSeTS into national and international policy frameworks could support the systematic identification and scaling of effective interventions, ensuring that efforts to reduce SIL are evidence-based, equitable, and sustainable. By promoting a standardized approach to intervention classification, ASSeTS has the potential to drive more coordinated, impactful, and scalable efforts to improve social connection and population health outcomes globally.

## Supplementary Information

Supplementary Material 1.

## Data Availability

All data and materials available in tables and Appendix.
