# Supplementary material for: ASSeTS: a systematic review and development of the World Health Organization’s classification system for social isolation and loneliness interventions
Source: Popul Health Metr. 2026 Mar 15;24:29. doi: 10.1186/s12963-026-00472-7 (PMC13104352; doi:10.1186/s12963-026-00472-7)
Supplement: Supplementary file 1 — Supplementary Material 1. [file 12963_2026_472_MOESM1_ESM.docx]

**Appendix**

**Appendix 1 |** PRISMA 2020 checklist

| **Section and Topic** | **Item #** | **Checklist item** | **Location where item is reported** |
| --- | --- | --- | --- |
| **TITLE** | | |  |
| Title | 1 | Identify the report as a systematic review. | Page 1 |
| **ABSTRACT** | | |  |
| Abstract | 2 | See the PRISMA 2020 for Abstracts checklist. | Page 1-2 |
| **INTRODUCTION** | | |  |
| Rationale | 3 | Describe the rationale for the review in the context of existing knowledge. | Page 4 |
| Objectives | 4 | Provide an explicit statement of the objective(s) or question(s) the review addresses. | Page 6 |
| **METHODS** | | |  |
| Eligibility criteria | 5 | Specify the inclusion and exclusion criteria for the review and how studies were grouped for the syntheses. | Page 7-8 |
| Information sources | 6 | Specify all databases, registers, websites, organisations, reference lists and other sources searched or consulted to identify studies. Specify the date when each source was last searched or consulted. | Page 7 |
| Search strategy | 7 | Present the full search strategies for all databases, registers and websites, including any filters and limits used. | Page 7/Table 1 |
| Selection process | 8 | Specify the methods used to decide whether a study met the inclusion criteria of the review, including how many reviewers screened each record and each report retrieved, whether they worked independently, and if applicable, details of automation tools used in the process. | Page 7-8 |
| Data collection process | 9 | Specify the methods used to collect data from reports, including how many reviewers collected data from each report, whether they worked independently, any processes for obtaining or confirming data from study investigators, and if applicable, details of automation tools used in the process. | Page 8 |
| Data items | 10a | List and define all outcomes for which data were sought. Specify whether all results that were compatible with each outcome domain in each study were sought (e.g. for all measures, time points, analyses), and if not, the methods used to decide which results to collect. | Page 8 |
|  | 10b | List and define all other variables for which data were sought (e.g. participant and intervention characteristics, funding sources). Describe any assumptions made about any missing or unclear information. | Page 9 |
| Study risk of bias assessment | 11 | Specify the methods used to assess risk of bias in the included studies, including details of the tool(s) used, how many reviewers assessed each study and whether they worked independently, and if applicable, details of automation tools used in the process. | Page 9 |
| Effect measures | 12 | Specify for each outcome the effect measure(s) (e.g. risk ratio, mean difference) used in the synthesis or presentation of results. | Page 9 |
| Synthesis methods | 13a | Describe the processes used to decide which studies were eligible for each synthesis (e.g. tabulating the study intervention characteristics and comparing against the planned groups for each synthesis (item #5)). | Page 9 |
|  | 13b | Describe any methods required to prepare the data for presentation or synthesis, such as handling of missing summary statistics, or data conversions. | Page 9-10 |
|  | 13c | Describe any methods used to tabulate or visually display results of individual studies and syntheses. | Page 9 |
|  | 13d | Describe any methods used to synthesize results and provide a rationale for the choice(s). If meta-analysis was performed, describe the model(s), method(s) to identify the presence and extent of statistical heterogeneity, and software package(s) used. | Page 9-10 |
|  | 13e | Describe any methods used to explore possible causes of heterogeneity among study results (e.g. subgroup analysis, meta-regression). | NA |
|  | 13f | Describe any sensitivity analyses conducted to assess robustness of the synthesized results. | NA |
| Reporting bias assessment | 14 | Describe any methods used to assess risk of bias due to missing results in a synthesis (arising from reporting biases). | NA |
| Certainty assessment | 15 | Describe any methods used to assess certainty (or confidence) in the body of evidence for an outcome. | Page 9 |
| **RESULTS** | | |  |
| Study selection | 16a | Describe the results of the search and selection process, from the number of records identified in the search to the number of studies included in the review, ideally using a flow diagram. | Page 10/Figure1 |
|  | 16b | Cite studies that might appear to meet the inclusion criteria, but which were excluded, and explain why they were excluded. | Table 2 |
| Study characteristics | 17 | Cite each included study and present its characteristics. | Page 10/Table 5 |
| Risk of bias in studies | 18 | Present assessments of risk of bias for each included study. | Page 11 |
| Results of individual studies | 19 | For all outcomes, present, for each study: (a) summary statistics for each group (where appropriate) and (b) an effect estimate and its precision (e.g. confidence/credible interval), ideally using structured tables or plots. | Page 14/Table 9 |
| Results of syntheses | 20a | For each synthesis, briefly summarise the characteristics and risk of bias among contributing studies. | Page 11/Table 7 |
|  | 20b | Present results of all statistical syntheses conducted. If meta-analysis was done, present for each the summary estimate and its precision (e.g. confidence/credible interval) and measures of statistical heterogeneity. If comparing groups, describe the direction of the effect. | Page 14/Table 9 |
|  | 20c | Present results of all investigations of possible causes of heterogeneity among study results. | NA |
|  | 20d | Present results of all sensitivity analyses conducted to assess the robustness of the synthesized results. | NA |
| Reporting biases | 21 | Present assessments of risk of bias due to missing results (arising from reporting biases) for each synthesis assessed. | NA |
| Certainty of evidence | 22 | Present assessments of certainty (or confidence) in the body of evidence for each outcome assessed. | Page 14/Table 9 |
| **DISCUSSION** | | |  |
| Discussion | 23a | Provide a general interpretation of the results in the context of other evidence. | Page 15 |
|  | 23b | Discuss any limitations of the evidence included in the review. | Page 16-17 |
|  | 23c | Discuss any limitations of the review processes used. | Page 17 |
|  | 23d | Discuss implications of the results for practice, policy, and future research. | Page 17-18 |
| **OTHER INFORMATION** | | |  |
| Registration and protocol | 24a | Provide registration information for the review, including register name and registration number, or state that the review was not registered. | Page 7 |
|  | 24b | Indicate where the review protocol can be accessed, or state that a protocol was not prepared. | No protocol published |
|  | 24c | Describe and explain any amendments to information provided at registration or in the protocol. | NA |
| Support | 25 | Describe sources of financial or non-financial support for the review, and the role of the funders or sponsors in the review. | Page 19 |
| Competing interests | 26 | Declare any competing interests of review authors. | Page 19 |
| Availability of data, code and other materials | 27 | Report which of the following are publicly available and where they can be found: template data collection forms; data extracted from included studies; data used for all analyses; analytic code; any other materials used in the review. | Tables, figure, and appendix files. |

*From:*  Page MJ, McKenzie JE, Bossuyt PM, Boutron I, Hoffmann TC, Mulrow CD, et al. The PRISMA 2020 statement: an updated guideline for reporting systematic reviews. *BMJ* 2021;372:n71. doi: 10.1136/bmj.n71

**Appendix 2 |** Sample size calculations

To estimate the required sample size needed to achieve an acceptable Fleiss’ Kappa score of ≥0.41 (moderate or stronger) (1) we used the ‘kappaSize’ function in R. We assumed five potential categories of outcomes, representative of the five different types of social isolation and loneliness interventions in the proposed classification system, not including ‘multicomponent’ or ‘other’ interventions:

*CI5Cats(kappa0, kappaL, kappaU=NA, props, raters, alpha)*

where ‘kappa0’ is the specified anticipated Kappa value, ‘kappaL’ and ‘kappaU’ are the specified expected lower and upper limits, ‘props’ are the anticipated prevalence rates for each category, ‘raters’ is the number of raters involved, and ‘alpha’ is the desired type 1 error rate (2). For all models, the ‘kappaL’ value is set as 0.41 to ensure an acceptable minimal Fleiss’ Kappa score of 0.41 (moderate) is achieved, the anticipated ‘kappa0’ value is set as 0.61 as this reflects the next level of agreement strength above moderate, and the anticipated ‘kappaU’ is set as NA to calculate using a one-sided confidence interval, as the upper limit was not relevant in this scenario (1).

For example, assuming equal outcome proportions across six raters:

*CI5Cats(kappa0=0.61, kappaL=0.41, kappaU=NA, props=c(0.2, 0.2, 0.2,0.2,0.2), raters=5, alpha=0.05)*

| **κ_0_^b^** | **κ_L_^b^** | **κ_U_^c^** | **π_C1_** | **π_C2_** | **π_C3_** | **π_C4_** | **π_C5_** | **Number of raters** | | | | |
| --- | --- | --- | --- | --- | --- | --- | --- | --- | --- | --- | --- | --- |
|  |  |  |  |  |  |  |  | **2** | **3** | **4** | **5** | **6** |
| 0.610 | 0.410 | NA | 0.200 | 0.200 | 0.200 | 0.200 | 0.200 | 27 | 15 | 12 | 11 | 11 |
| 0.610 | 0.410 | NA | 0.500 | 0.125 | 0.125 | 0.125 | 0.125 | 32 | 18 | 14 | 11 | 11 |
| 0.610 | 0.410 | NA | 0.500 | 0.300 | 0.100 | 0.050 | 0.050 | 36 | 20 | 15 | 12 | 11 |

κ_0_: specified anticipated Kappa value; κ_L/U_: specified expected lower and upper limits; π_C1-C5_: category prevalence rates

1. Landis JR, Koch GG. The measurement of observer agreement for categorical data. Biometrics. 1977;33(1):159-74.
2. Rotondi MA, Donner A. A confidence interval approach to sample size estimation for interobserver agreement studies with multiple raters and outcomes. J Clin Epidemiol. 2012;65(7):778-84.
